# Enhancing Smart Sensor Tag Sensing Performance-Based on Modified Plasma-Assisted Electrochemical Exfoliated Graphite Nanosheet

**DOI:** 10.3390/polym14235067

**Published:** 2022-11-22

**Authors:** Tzu-Hsuan Lin, Alan Putranto, Yan-Ting Wang, Qing-Hao Yang, Ren-Jang Wu, Chia-Hao Liu, Che-Kuan Lin, Murthy Chavali

**Affiliations:** 1 Department of Civil Engineering, National Central University, Taoyuan 32011, Taiwan; 2 Department of Civil Engineering, Ketapang State Polytechnic, Ketapang 78813, Indonesia; 3 Department of Applied Chemistry, Providence University, Taichung 43301, Taiwan; 4 Office of the Dean (Research) and Department of Chemistry, Faculty of Science Technology, Alliance University (Central Campus), Chandapura-Anekal Main Road, Bengaluru 562106, Karnataka, India; 5 Materials Department, NTRC-MCETRC, Guntur District, Tenali 522201, Andhra Pradesh, India

**Keywords:** modified RFID, nanosheet coating layer, non-destructive test, seepage detection, concrete structure, sensitivity performance

## Abstract

Water that penetrates through cracks in concrete can corrode steel bars. There is a need for reliable and practical seepage sensing technology to prevent failure and determine the necessary maintenance for a concrete structure. Therefore, we propose a modified plasma-assisted electrochemical exfoliated graphite (MPGE) nanosheet smart tag. We conducted a comparative study of standard and modified RFID smart tags with sensor technology for seepage detection in concrete. The performance of both smart tags was tested and verified for seepage sensing in concrete, characterized by sensor code and frequency values. Seepage was simulated by cracking the concrete samples, immersing them for a designated time, and repeating the immersing phase with increasing durations. The test showed that the modified smart tag with 3% MPGE and an additional crosslinking agent provided the best sensitivity compared with the other nanosheet compositions. The presence of 3D segregated structures on the smart tag’s sensing area successfully enhanced the sensitivity performance of seepage detection in concrete structures and is expected to benefit structural health monitoring as a novel non-destructive test method.

## 1. Introduction

Water is an agent of deterioration in both physical and chemical processes, leading to the degradation of concrete structures [1]. Water that penetrates through cracks in concrete can cause corrosion, and multiple methods can be used to monitor the presence of water in concrete [2,3,4]. A contactless structure monitoring system using radio frequency identification (RFID) technology optimized for seepage sensing in concrete structures is relatively unexplored. The flexibility of RFID tags could allow for structure performance monitoring by embedding smart tags into the concrete.

A prior study has investigated the performance of smart tags encased in two different types of deep case protections to detect seepage in concrete and determine the sensitivity of detection for designated conditions (dry and water deposition) around the sensing area [5]. Researchers determined that sensor code-based RFID technology provided the best indication of seepage. Moreover, sensor codes for smart tags with different thicknesses of polydimethylsiloxane (PDMS) film protection were defined for the dielectric properties of electrolyte solutions at different concentrations [6]. As a result, the sensor code value decreased as water content, or any aqueous electrolyte solution increased around the sensing area. Over the past decade, studies have focused on implementing RFID technology for structural health monitoring (SHM) systems. The SHM system with non-destructive test (NDT) techniques for assessing structures plays a significant role in the construction industry. In 2013, researchers developed a crack damage sensor-based RFID technology for SHM application [7], specifically for the investigation and monitoring of cracks in metallic structures based on laboratory tests with multiple sensor array configurations [8]. Crack damage-based RFID technology had indirect seepage-induced identification and was ineffective in monitoring corrosion indicators in concrete structures. Therefore, research has focused on embedded RFID technology for monitoring purposes [9,10].

A passive wireless concrete humidity monitoring system in the curing phase, with a tag embedded at an 8 cm depth, showed reliable communication performance [11]. A suitable design of the ultra-high frequency (UHF) RFID tag antenna embedded in concrete has been investigated. The antenna had an absolute gain of 3.6 dBm and a read range of about 12.8 m [12]. A concept of long-term monitoring [13] with an RFID sensor system embedded in concrete structures was introduced in 2017. They conducted several encased types of high frequency (HF) and UHF RFID antennas and observed that polyvinylchloride shows excellent results after 14 days in NaOH solution. A comprehensive study of transmission characteristics of RFID sensor systems embedded in concrete structures with different embedding depths for both HF and UHF RFID has also been investigated [14]. However, researchers have not considered the real problem of existing structures, such as underground structures (tunnels), bridges, and coastal structures. These structures may experience cracks due to external load and environmental conditions that lead to water penetration into the concrete and change the reading measurement parameters. To date, few researchers have addressed this problem [4,5]. It is crucial to identify the excessive amount of water in existing reinforced concrete (RC) structures, which is responsible for the corrosion of reinforcement steel and is reported as the primary cause of 70–90% of practical cases of premature deterioration of RC structures [15]. A practical and reliable assessment method for seepage monitoring in existing concrete structures with RFID technology should be established.

Although not specifically designed for seepage sensing applications, some researchers developed and modified an existing standard battery-free wireless RFID sensor tag for sensing purposes and proposed different methods. They modified the tag antenna coated with graphene oxide (GO) and measured its relative dielectric permittivity for various humidity conditions [16]. Similarly, microscale capacitive humidity sensors were also coated with GO film [17]. Indeed, GO is attractive due to these combined advantages, as well as other promising properties, such as high surface area, oxygenated functional groups, and excellent thermal stability [18,19,20]. Adopting GO as a nanocomposite material, we introduce a modified plasma-assisted electrochemical exfoliated graphite (MPGE) nanosheet coated on the smart tag’s sensing area for seepage sensing sensitivity performance in concrete. The modified plasma-assisted electrochemical exfoliated graphite (MPGE) nanosheet production method involves related chemical reduction and physical procedures. The chemical reduction is connected to the modified MPEG resistance value, and the peeling method was used for the plasma-assisted electrochemical exfoliated system [21]. Another research field of molecular computing, such as the Fukui function application, and modified and exfoliated graphene, could help the degree of electronic transition on the surface of graphene, which is widely used in biochemical medicine [22,23,24].

This research conducts a comparative study between standard smart tags and modified smart tags for seepage detection systems as a non-destructive test method based on RFID technology. We proposed a modified plasma-assisted electrochemical exfoliated graphite (MPGE) nanosheet smart tag, which is described as a modified smart tag for the rest of this paper. Several compositions of the modified smart tag are investigated. The sensitivity-sensing performance of these smart tags are then discussed.

## 2. Principle of Seepage Sensing Mechanism

**Figure 1:**
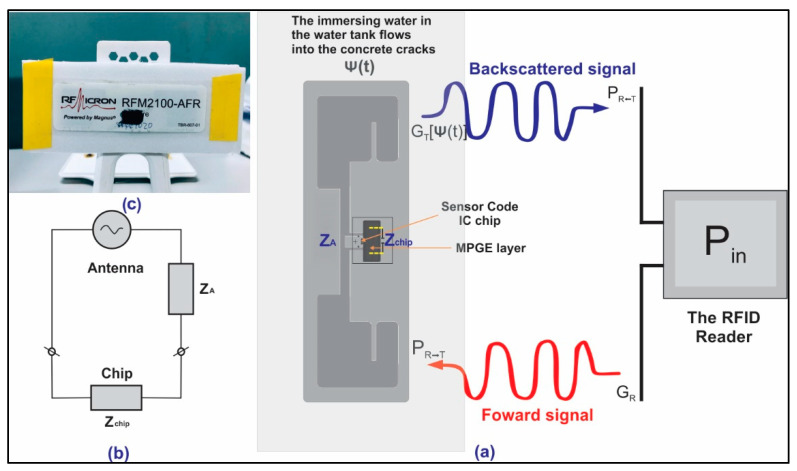
(**a**) Schematic principle of MPGE smart sensor system; (**b**) the equivalent circuit of the MPGE smart tag; and (**c**) coated MPGE layer on smart tag’s interdigitated capacitor.

The sensing mechanism of this work comprised a synthesis of MPGE, optimization, and characterization. After characterization, the MPGE coating was applied to the substrate with the interdigitated sensing area on a passive smart tag. In the sensor-code integrated circuits (ICs), the capacitor bank had 32 capacitance states represented by a 5-bit sensor code that was the tuning setting. The ICs work as signal converters and transmit the electromagnetic (EM) waves back to the RFID reader. The sensor code provides a measure of the tag antenna’s impedance. The antenna’s impedance changes between readings and the sensor code changes as the chip adapts to the bank of capacitors and matches the antenna impedance at the time of each reading. The change in sensor code then indicates a change in the antenna impedance. If the tag antenna is designed to respond to a change in the environment, such as changes in moisture, the sensor code could consequently be used to quantify these environmental changes. A schematic of the MPGE smart sensor system is illustrated in Figure 1.

The backscatter signal-based RFID sensor system was proposed to monitor physical changes within the concrete structure. In this case, a change in physical properties was denoted as ψ(*t*). A classical simplified free-space interaction was introduced in a previous study [25]. It has been used to explain the wireless sensing mechanism for any changing physical properties (i.e., chemical, local physical, or geometrical parameters of the tag’s surrounding environment). Therefore, the following equations, Equations (1) and (2) describe the power collected at the sensor’s microchip and the power backscattered by the RFID tag toward the RFID reader, respectively.

(1) $$P_{R\to T}\left[\psi \right]={\left(\frac{\lambda_{0}}{4\pi d}\right)}^{2}P_{in}G_{R}\left(\theta ,\varphi \right)G_{T}\left[\psi \right]\left(\theta ,\varphi \right)\tau \left[\psi \right]\eta_{p}$$

(2) $$P_{R\leftarrow T}\left[\psi \right]=\frac{1}{4\pi }{\left(\frac{\lambda_{0}}{4\pi d^{2}}\right)}^{2}P_{in}G_{R}^{2}\left(\theta ,\varphi \right)\eta_{p}^{2}rcs_{T}\left[\psi \left(\theta ,\varphi \right)\right]$$

where *λ*_0_ is the wavelength; *d* is the distance between the smart tag and RFID reader; *P_in_* and *G_R_* are denoted for the power entering and gain obtained by the RFID reader’s antenna, respectively; (*θ*, *ϕ*) is the direction; *G_T_*[*ψ*] is the gain in the tag’s antenna during the process at specific realization; *η_P_* is denoted for the polarization mismatch between the smart tag and RFID reader, and *τ*[*ψ*] is denoted for the power transmission coefficient of the smart tag. The tag’s power-transmission coefficient can be calculated using Equation (3). Meanwhile, *rcs_T_* in Equation (2) is the radar cross-section of the tag, which is related to the modulation impedance and can be calculated using Equation (4). Furthermore, *Z*_mod_ in the following equation is denoted for the microchip for encoding low and high digital states.

(3) $$\tau \left[\psi \right]=\frac{4R_{chip}R_{a}\left[\psi \right]}{{\left[Z_{chip}+Z_{a}\left[\psi \right]\right]}^{2}}$$

(4) $$rcs_{T}\left[\psi \right]=\frac{\lambda_{0}^{2}}{4\pi }G_{T}^{2}\left[\psi \right]\left(\theta ,\varphi \right){\left(\frac{2R_{a}\left[\psi \right]}{\left|Z_{\mathrm{mod}}+Z_{a}\left[\psi \right]\right|}\right)}^{2}$$

The backscattered power in the received signal strength indicator (RSSI) is presumed to correspond to the modulating state, as *Z*_mod_ = *Z_chip_*. The abovementioned formula introduces a classical RFID sensing mechanism for indirect type measurement. In this study, the term “smart” in the smart sensor tag consisted of a measurement parameter (sensor code) that is sensitive to moisture change and could be used as a direct measurement. This parameter was then used as the seepage indicator. The sensor code’s damage index (*DI_SC_*), which consists of sensor code values, was introduced. The sensor code’s damage index value is the difference between the values representing the sensor code measured at an initial state and after the specimen is soaked. Several reading measurements were conducted and calculated as an average value of the sensor code to determine the sensor code’s damage index, as shown in Equation (5) below.

(5) $$DI_{SC}=SC_{i}-SC_{S}$$

To accommodate different read range measurements in seepage sensing, a total damage index (*TDI*) was introduced. The *TDI* is the sum of damage index values divided by the total number of measured read ranges, which is given in Equation (6) below.

(6) $$TDI=\frac{\sum_{j-1}^{n}DI_{j}}{n}$$

## 3. Materials and Method

The material used in the synthesis process is described as follows: the poly butyl acrylate (PBA, M35, waterborne type, solid content of 50 ± 3 wt%) and the isocyanate crosslinking agent (UC-883CW, waterborne type, solid content of 80 ± 3 wt%) were used as the main material. The plasma-assisted electrochemical exfoliation of graphite (PGE) in the liquid phase (with a solid content of ≈1%) was provided by the laboratory of the National Chung-Shan Institute of Science Technology, Taiwan. The hydrazine hydrate solution (ALF 16651, purity ≥ 99%) was used as a modifier for the plasma-assisted electrochemical exfoliation of graphite.

**Figure 2:**
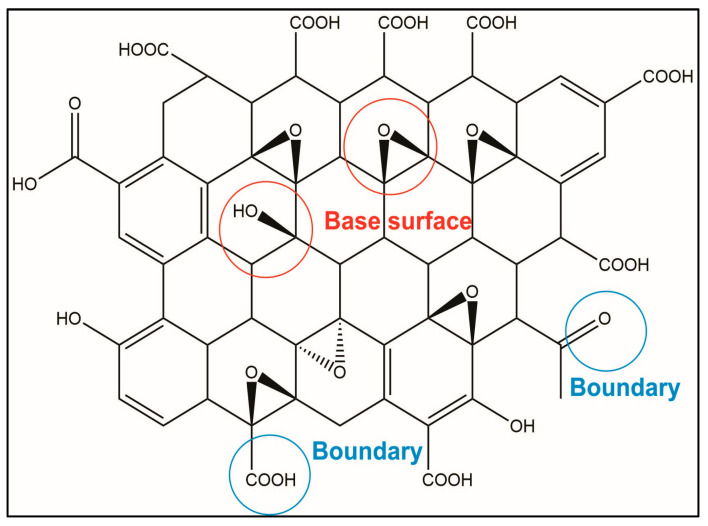
Schematic reaction mechanism of PGE reduction by hydrazine hydrate.

In this study, we modify the PGE, which, before modification, is a graphene material with oxidizing groups bonded on the surface. However, after the PGE is modified, the graphene oxide forms reduced graphene (modified PGE, MPGE). The difference between graphene oxide and reduced graphene is that the basal plane and edge of graphene oxide contain many oxygen-containing functional groups (oxygen functional groups). The electronegativity of oxygen atoms hinders the transfer efficiency of electrons on graphene. The upper graphene oxide structure is not a complete plane, which can also cause difficulties in electron transfer. The structure of the molecule is shown in Figure 2.

To overcome the cohesive stacking issue, the PGE (1.02 mg/mL) was vigorously stirred for 48 h, then aliquoted into 100 mL vials and fully exfoliated using sonication at 400 W for 2 h. Next, one 100 mL aliquot of PGE with 5 g of hydrazine hydrate was added into a 500 mL round flask. The PGE was reduced and modified by hydrazine hydrate at 95 °C for 6 h with magnetic stirring at 400 rpm to increase both the collision probability of the chemical reaction and the reaction force, and simultaneously supply the energy required for the chemical reaction. The resultant modified PGE was passed through a membrane filter and simultaneously washed with deionized water several times until the residual hydrazine hydrate was completely removed. Finally, the modified PGE was dried in a vacuum oven at 70 °C. PGE after modification by hydrazine hydrate is henceforth abbreviated as MPGE for simplicity.

Synthesis of the MPGE layer consisted of four different processes:

- Producing 1% MPGE was done by stirring 20 g PBA and 0.2 g MPGE power together in a container at 60 °C for 10 min, until the liquid turned green.
- The heater was turned off and the solution was stirred for an additional 20 min, followed by dispersing and stirring at 40 °C for 6 h.
- An amount of 0.2 g of a crosslinking agent was added and the solution was stirred at 40° for an additional 2 h. At this time, the RFID was coated with the liquid using a dropper and placed in the oven for drying.
- Additionally, 1% MPGE was created using the same process, but without adding the crosslinking agent. The above-mentioned process was repeated to create 3% MPGE, both with and without a crosslinking agent.

**Figure 3:**
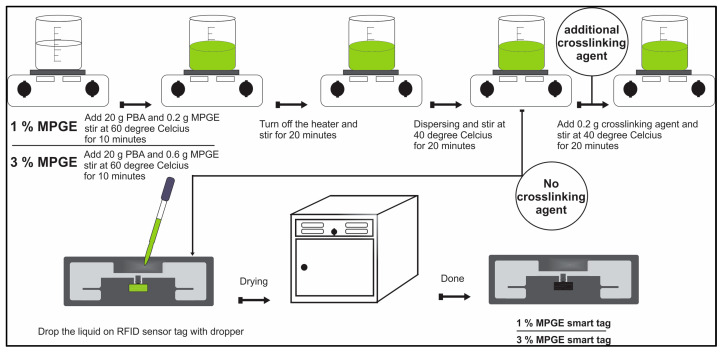
Schematic synthesis of MPGE smart tag with and without a crosslinking agent.

**Figure 4:**
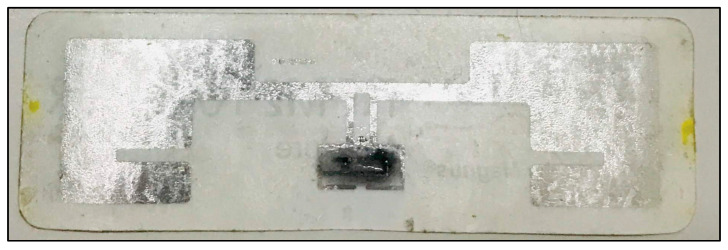
Photograph of MPGE smart tag.

The schematic synthesis process can be seen in Figure 3. The final product of the synthesizing and coating process is shown in Figure 4.

## 4. Experiment Design

**Figure 5:**
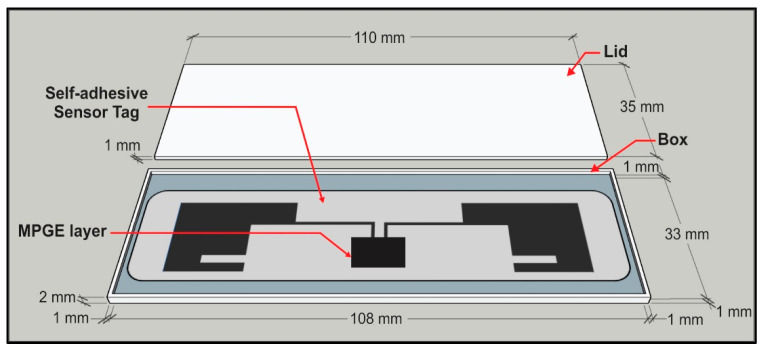
A 3 mm-deep 3D-printed case.

**Figure 6:**
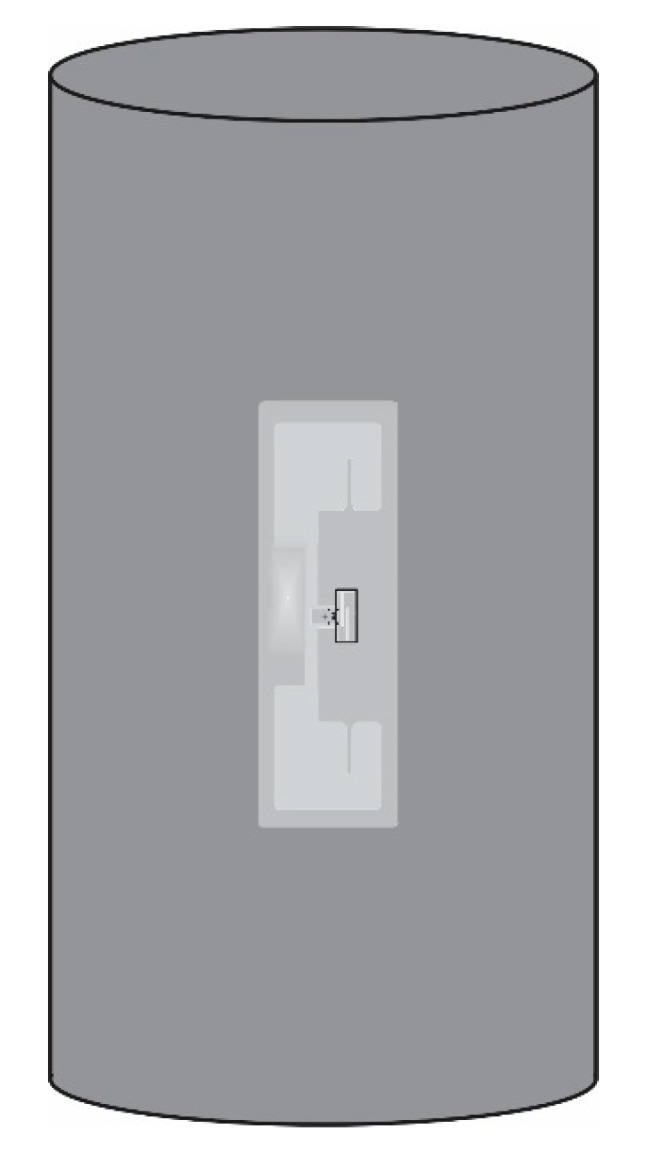
Schematic diagram of the 3D-printed protection of smart sensor tag embedded in cylindrical concrete.

In this research, the concrete samples were designed to have a different embedded RFID sensor tag. There were seven cylinder concrete samples. The samples were 100 mm in diameter and 200 mm tall. Four cylindrical concrete specimens were embedded with four different coated RFID smart tags: (1) 1% MPGE, (2) 1% MPGE with a crosslinking agent, (3) 3% MPGE, and (4) 3% MPGE with a crosslinking agent. To ensure the performance and protect the smart tags before embedding them in the concrete cylinder, each MPGE smart tag was encased with a 3 mm-deep 3D-printed case made from PLA, as shown in Figure 5. Meanwhile, another three cylindrical concrete specimens were embedded with standard smart tags with the same case protection. For further detailed information, please see Table 1. The schematic diagrams of the 3D-printed cases that protect the RFID sensor tag embedded in cylindrical concrete are shown in Figure 6.

**Figure 7:**
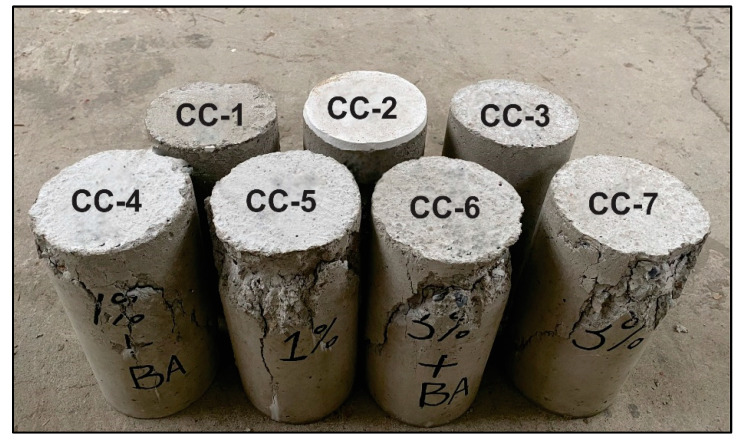
Photograph of cylindrical concrete specimens after applied load.

In this experiment, we investigated the effect of additional MPGE layers on sensing areas for seepage sensing sensitivity inside the concrete. As the corrosion indicator, the presence of excess water inside the concrete would lead to premature deterioration. Water usually penetrates concrete through cracks. Therefore, the concrete samples were designed for two conditions, before damage (before applied load) and after damage (after applied load). The cracks were created by applying a load to the cylinders using a compression test machine. The specimens after the load was applied are shown in Figure 7.

The seepage mechanism was simulated by fully immersing the samples in water for 24, 48, or 72 h. First, both concrete specimens with MPGE smart tags and standard smart tags were dried at 70 °C for 24 h to ensure the measurements had the same initial conditions after curing for 28 days. Then, each specimen was measured twice, before damage, and after damage, and the read range measurement was designed for 50, 100, and 150 cm away from the RFID reader. The concrete specimens were further investigated after being fully immersed for the designated time. Then, each specimen was lifted for measurement purposes. The sensor-code values were recorded in every read range and immersion time. The utilized RFID reader followed the EPCglobal Gen2 (ISO 18000-6C) air protocol and operates in the frequency range of 902–928 MHz. Both power over Ethernet (PoE) and Wi-Fi reader options were feasible, but PoE was chosen for transmission stability. The radio frequency (RF) transmission power was 30 dBm throughout the experiment, which was the maximum output power with an integrated antenna. A dipole antenna-type tag was used in this research. Meanwhile, the antenna reader was a fully integrated 8.5 dBic circular-polarized antenna and a Reverse Polarity-Threaded Neill Concelman (RP-TNC) connector that supported an optional secondary external antenna. The read ranges varied from 50 to 150 cm, and the specimen was moved away from the RFID reader in 50 cm increments, in which the measurement process had a 60 s read-time in each position.

## 5. Results and Discussion

**Figure 8:**
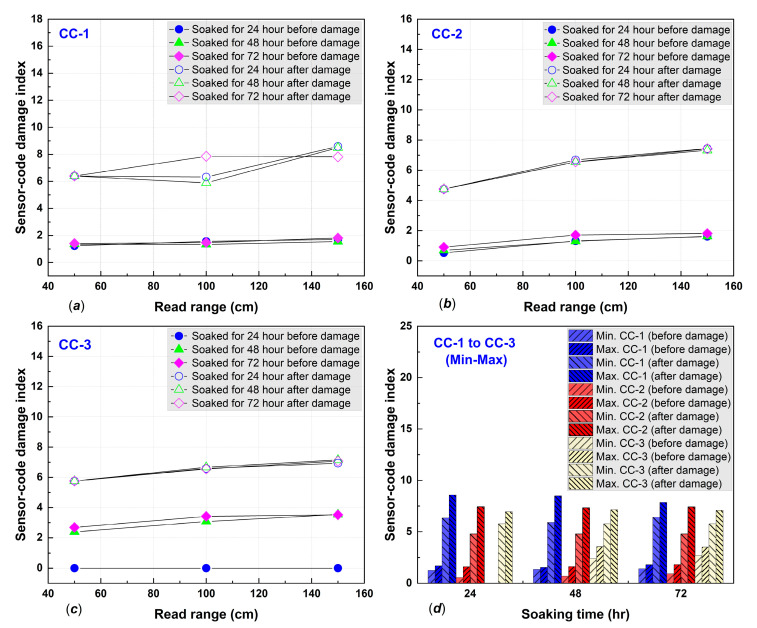
Damage indices of (**a**) CC-1, (**b**) CC-2, (**c**) CC-3, and (**d**) minimum and maximum of before–after damage.

**Figure 9:**
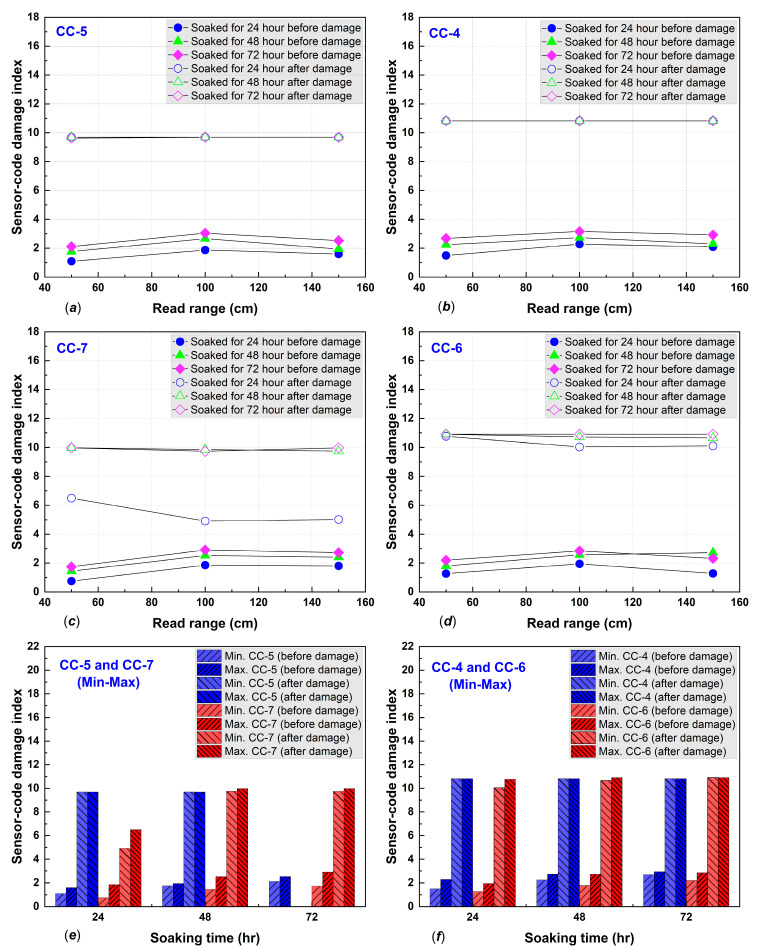
Damage indices of (**a**) CC-5, (**b**) CC-4 (**c**) CC-7, (**d**) CC-6, (**e**) minimum and a maximum of CC-5 and CC-7 before–after damage, and (**f**) minimum and a maximum of CC-4 and CC-6 before–after damage.

The damage indices were calculated using (5), and the results are shown in detail in Figure 8 and Figure 9. Damage indices (DIs) were inconsistent for both standard and MPGE smart tags alongside increasing immersion times. The inconsistency of DIs was due to variations in the crack width and pattern of the concrete specimens. We also found that the minimum and maximum DIs for both standard and MPGE smart tags increased from before damage to after damage, as shown in Figure 8d and Figure 9e–f. Due to the inconsistency of damage indices in every read range, a statistical approach was required to determine the seepage mechanism. The seepage mechanism was then identified using Equation (6) for each immersion time. The seepage mechanism was previously investigated and reported that seepage happened when the TDI after damage was higher than before damage, along with an increase in immersed time [5].

**Figure 10:**
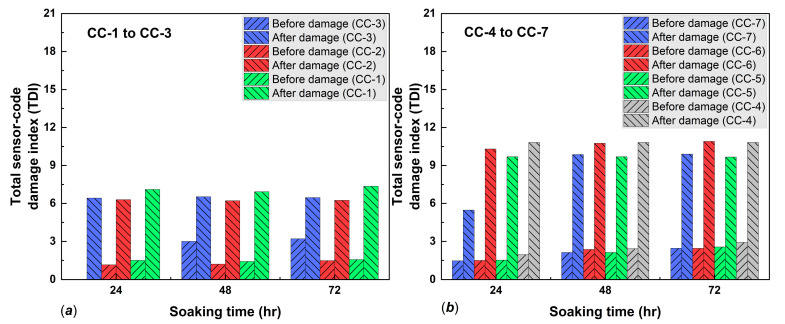
Seepage indicator based on TDI of (**a**) standard smart tags and (**b**) MPGE tags.

In the present study, the seepage mechanism detected with both standard and MPEG smart tags were examined through the total sensor-code damage index. The TDI for CC-1 after 24 h immersion was 1.5 before damage and 7.1 after damage; after 48 h, TDI was 1.4 before and 6.9 after damage; and after 72 h, TDI was 1.5 and 7.3, respectively. The CC-2 specimen had before-damage TDIs of 0.9, 1.0, and 1.3 and TDIs after damage were 6.3, 6.2, and 6.2, for 24, 48, and 72 h of immersion, respectively. The CC-3 specimen TDIs before damage were 0.0, 3.0, and 3.2 and after-damage TDIs were 6.4, 6.5, and 6.4, after immersing for 24, 48, and 72 h, respectively. The details of the results from the three concrete specimens with embedded standard smart tags are shown in Figure 10a and confirm seepage. All the TDIs were higher after damage than before damage.

Other concrete specimens with embedded 1% MPGE smart tags (CC-5) had TDIs before damage of 1.5, 2.1, and 2.5 for 24, 48, and 72 h of immersion, respectively, and after-damage TDIs were stable at 9.6. On the other hand, 1% MPGE with the crosslinking agent (CC-4) had TDIs before damage of 1.9, 2.4, and 2.9 for 24, 48, and 72 h of immersion, respectively, and were stable at 10.8 after damage. Meanwhile, the 3% MPGE (CC-7) had TDIs before damage of 1.4, 2.1, and 2.4, and TDIs of 5.4, 9.8, and 9.9 after damage, for 24, 48, and 72 h of immersion, respectively. The other 3% MPGE with the crosslinking agent (CC-6) had TDIs before damage of 1.5, 2.3, and 2.4, and TDIs after damage of 10.3, 10.7, and 10.9, for 24, 48, and 72 h of immersion, respectively. The TDI of the MPGE smart tags also confirmed seepage (Figure 10b).

**Figure 11:**
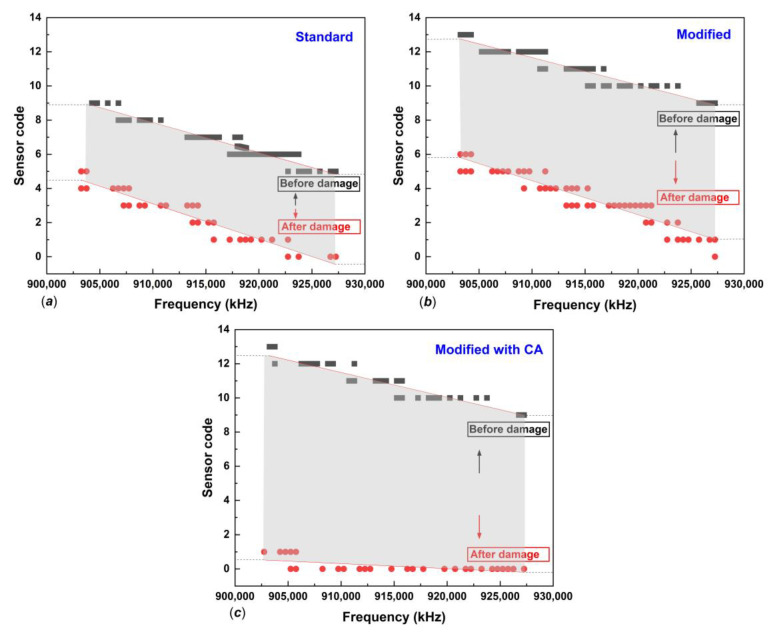
The sensitivity sensing performance of (**a**) standard smart tag, (**b**) MPGE tag, and (**c**) MPGE tag with a crosslinking agent.

**Figure 12:**
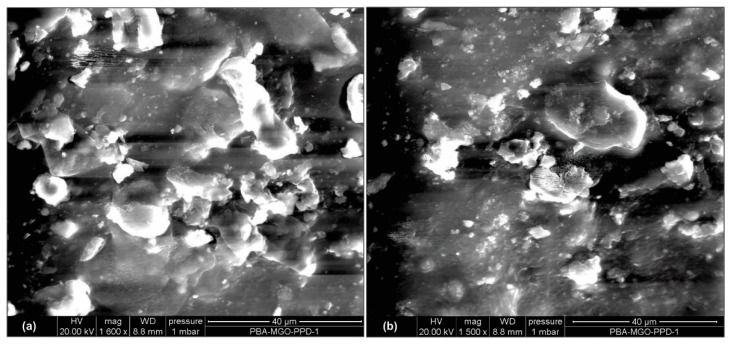
Scanning electron microscopy (SEM) of (**a**) 1% MPGE with a crosslinking agent and (**b**) 1% MPGE.

**Figure 13:**
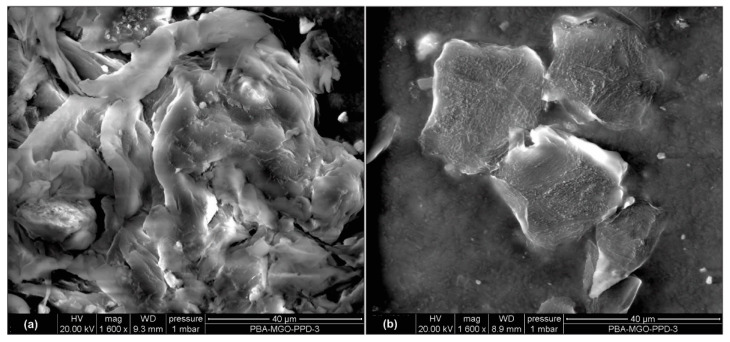
Scanning electron microscopy (SEM) of (**a**) 3% MPGE with a crosslinking agent and (**b**) 3% MPGE.

In subsequent analysis, the sensing sensitivity performance of the two types of tags for seepage detection in the concrete structure was examined through the relation between sensor code and frequency. Experimental single reads of tags embedded in concrete, immersed for 24 h, at a 50 cm measurement distance, indicated that seepage sensing sensitivity could be identified from sensor code values in the frequency range of 902–928 MHz with a decreasing pattern. The 3% MPEG and standard smart tag showed a pattern of decreasing sensor code values with moderate and standard sensitivity, respectively, as shown in Figure 11a,b. The smart tag with 3% MPGE with a crosslinking agent coating modification on the interdigitated capacitor area provided the highest sensitivity, with a significant change in sensor code values, as shown in Figure 11c. The MPGE coating layer on the tag’s sensing area successfully enhanced seepage sensing sensitivity, mainly because the homogeneous dispersion of MPGE with PBA successfully created 3D segregated network structures due to the amide bond, forming an intact connection between MPGE nanosheets [21]. The construction of 3D segregated network structures in the coating layer at the sensing area, as shown in scanning electron microscopy (SEM) images in Figure 12 and Figure 13, enhanced the electrical and mechanical properties of the tag’s sensing area. Thus, MPGE smart tags with the additional crosslinking agent were more sensitive to environmental change, especially seepage in the concrete structure.

The graphene coating nanosheet layer in this study was found to have potential for application in a non-destructive test based on RFID technology, and graphene also showed potential application in different areas [26,27,28]. Graphene with a 2D layer with sp^2^ orbitals of carbon atoms arranged as a honeycomb structure has also been explored as a nanofiller in various applications [29]. During synthesis, we developed a simple and effective way to prepare graphene-filled nanocomposites (GNs) with a 3D segregated network structure, which played a crucial part in reinforcing the electrical conductivity and mechanical properties of GNs. We found that the conductivity of the modified functional group of MPGE was better than that of only adding PGE. The oxygen atom in the reduced structure of the graphene made the surface of the graphene flatter, which was conductive to the electronic transition in the sp^2^ electronic orbital. Moreover, the amide bond formation during the preparation of MPGE (PBA) nanocomposites was conductive to constructing 3D segregated networks. Therefore, the synthesis of MPGE nanocomposites on smart tags provides an innovative and efficient way to enhance their sensitivity.

## 6. Conclusions

This study was carried out using standard smart tags and customized smart tags for seepage detection systems to conduct a non-destructive test procedure based on RFID technology. We have presented a modified smart tag called a modified plasma-assisted electrochemical exfoliated graphite (MPGE), a nanosheet smart tag described as a modified smart tag. Several synthesis compositions for the modified smart tag were developed. The sensitivity-sensing performance of these smart tags was then discussed.

In line with developing a smart seepage detection system, this study successfully modified and enhanced the seepage sensing sensitivity of non-destructive tests using a nanosheet material. The RFID sensor tag with the MPGE layer and crosslinking agent provided the highest sensitivity for detecting seepage. The construction of 3D-segregated structures of MPGE layers coated on sensing areas successfully enhanced the performance of RFID sensor technology.

We emphasize that with a suitable protective encasement, the MPGE smart tags can work more effectively and are highly sensitive compared with standard smart tags for sensing environmental changes, particularly seepage within concrete structures. This technology has other advantages, such as low cost and easy deployment. Due to its flexibility and being maintenance-free, this technology shows high potential for long-term structure monitoring applications requiring embedding into concrete structures. The proposed work, however, is limited by the time-consuming synthesis and coating process of nanosheet material. Nevertheless, MPGE smart tags offer a practical solution, particularly for the deployment of sensors in monitoring concrete structures subject to seepage or leakage.

## Figures and Tables

**Table 1:** Comparison of material properties of each specimen.

Specimen Code	Density (kg/m^3^)	Smart Tag Type	Chemical Compositions		Chemical Addition	Cased Type
			MPGE (g)	PBA (g)	Crosslinking Agent (g)	
CC-1	2125	Standard	-	-	-	3D-printed PLA case
CC-2	2159	Standard	-	-	-	3D-printed PLA case
CC-3	2147	Standard	-	-	-	3D-printed PLA case
CC-4	2087	MPGE	0.2	20	0.2	3D-printed PLA case
CC-5	2087	MPGE	0.2	20	-	3D-printed PLA case
CC-6	2080	MPGE	0.6	20	0.2	3D-printed PLA case
CC-7	2083	MPGE	0.6	20	-	3D-printed PLA case

*Note*. Dashes indicate that there is no chemical material in the synthesis process.

## Data Availability

Data presented in this study are available upon request from the corresponding authors.
